# Supplementary material for: Isolation and Genomic Analysis of 3-Chlorobenzoate-Degrading Bacteria from Soil
Source: Microorganisms. 2023 Jun 28;11(7):1684. doi: 10.3390/microorganisms11071684 (PMC10383586; doi:10.3390/microorganisms11071684)
Supplement: Supplementary file 1 [file microorganisms-11-01684-s001.zip › microorganisms-2413234-supplementary.pdf]

Supplemental Materials

Isolation and genomic analysis of 3-chlorobenzoate-degrading bacteria from soil

Ifat Ara[1], Ryota Moriuchi[2], Hideo Dohra[2,3], Kazuhide Kimbara[1], Naoto Ogawa[4], Masaki Shintani[1,5,6,7*]

[1]Department of Environment and Energy Systems, Graduate School of Science and Technology, Shizuoka University, 3-5-1, Johoku, Naka-ku, Hamamatsu, Shizuoka, 432-8561, Japan, ara.ifat.18@shizuoka.ac.jp, kimbara.kazuhide@shizuoka.ac.jp

[2]Functional Genomics Section, Shizuoka Instrumental Analysis Center, Shizuoka University, 836 Oh-ya, Suruga-ku, Shizuoka, 422-8529, Japan, moriuchi.ryota@shizuoka.ac.jp, dora.hideo@shizuoka.ac.jp

[3]Department of Science, Graduate School of Integrated Science and Technology, Shizuoka University, 836 Oh-ya, Suruga-ku, Shizuoka, 422-8529, Japan

[4]Department of Agriculture, Graduate School of Integrated Science and Technology, Shizuoka University, 836 Oh-ya, Suruga-ku, Shizuoka, 422-8529, Japan, ogawa.naoto@shizuoka.ac.jp

[5]Department of Engineering, Graduate School of Integrated Science and Technology, Shizuoka University, 3-5-1, Johoku, Naka-ku, Hamamatsu, Shizuoka, 432-8561, Japan;

[6]Japan Collection of Microorganisms, RIKEN BioResource Research Center, 3-1-1, Koyadai, Tsukuba, Ibaraki, 305-0074, Japan

[7]Research Institute of Green Science and Technology, Shizuoka University, 3-5-1, Johoku, Naka-ku, Hamamatsu, Shizuoka, 432-8561, Japan

***Corresponding authors: shintani.masaki@shizuoka.ac.jp; Tel.: +81-53-4781181**

a)

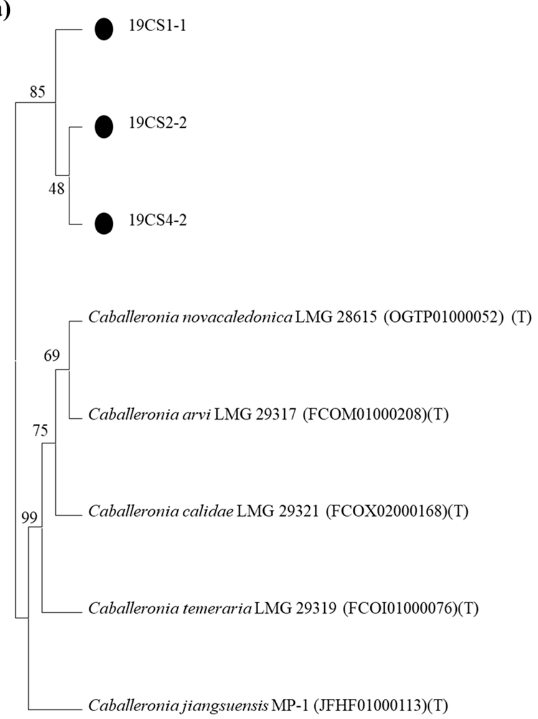

b)

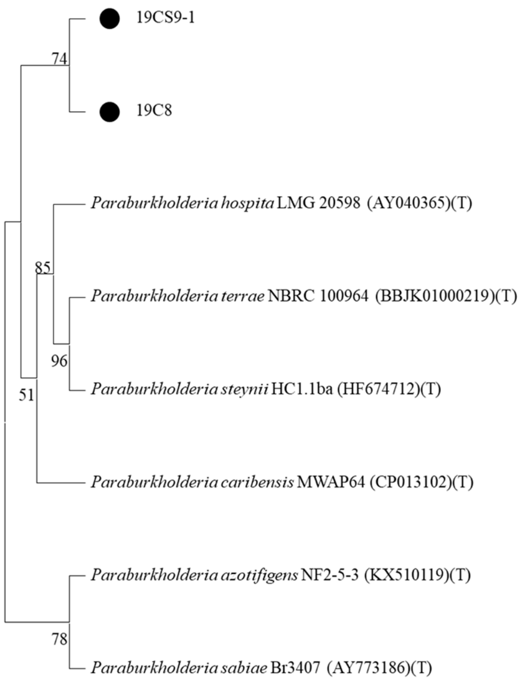

c)

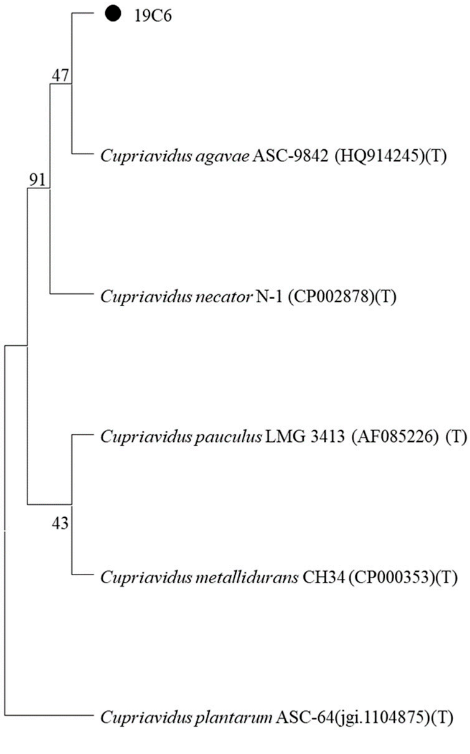

Figure S1:
Dendrogram showing the relationship of the 16S rRNA gene sequence of strain isolates (19CS4-2,19CS1-1,19CS2-2, 19CS9-1,19C8,19C6) with those reported for species of the genus *Caballeronia* (a)*, Paraburkholderia* (b)and *Cupriavidus* (c). The phylogenetic tree was constructed using the neighbor-joining method. Bootstrap percentages, based on 1000 replicates, are given at the nodes. GenBank accession numbers used for phylogenetic analysis are in parentheses. The obtained isolated bacteria are indicated by round shape marker.

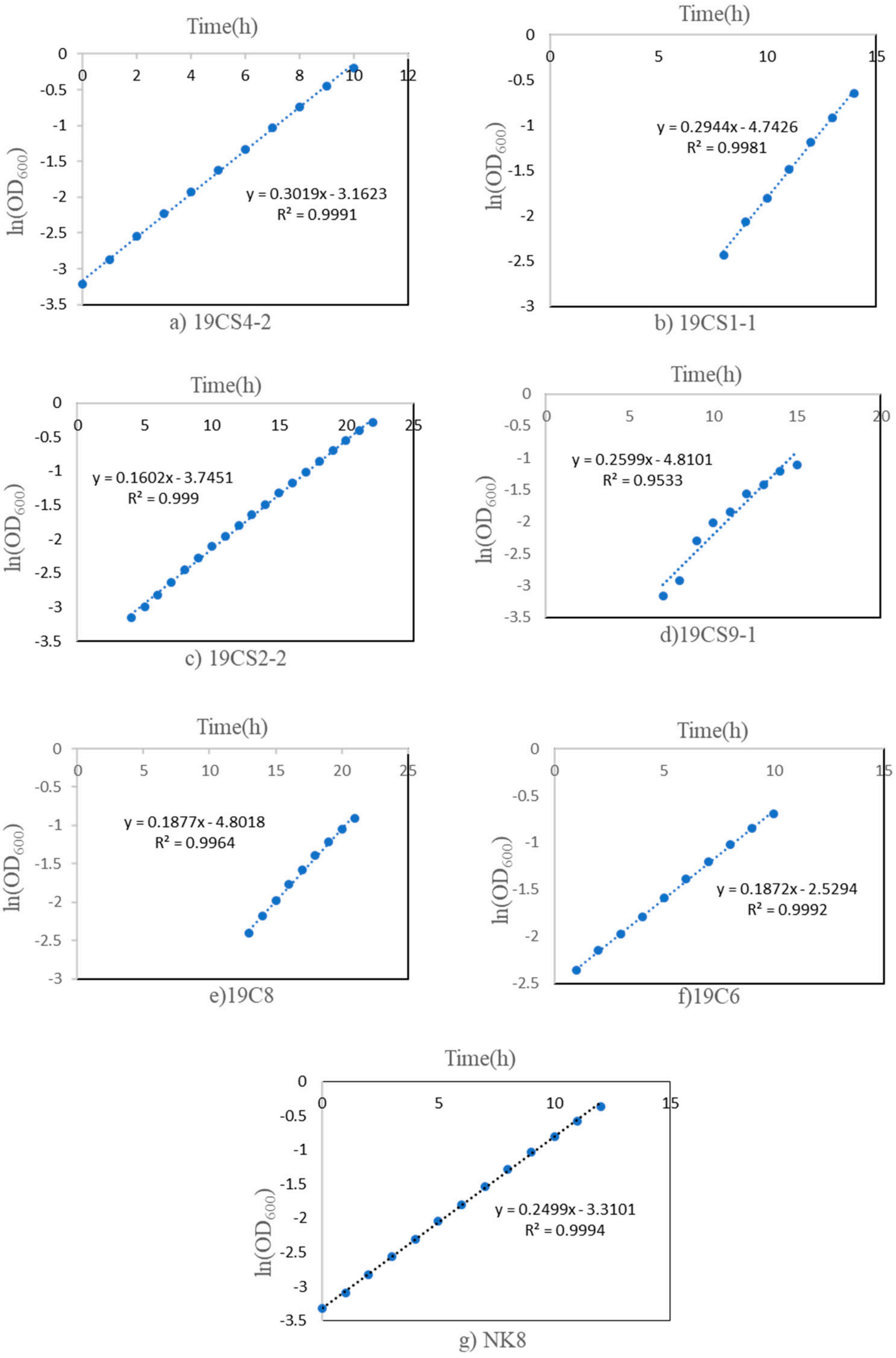

Figure S2: Growth kinetic results of isolates a) 19CS4-2, b) 19CS1-1, c) 19CS2-2, d) 19CS9-1, e) 19C8, f) 19C6 and reference strain g) NK8.

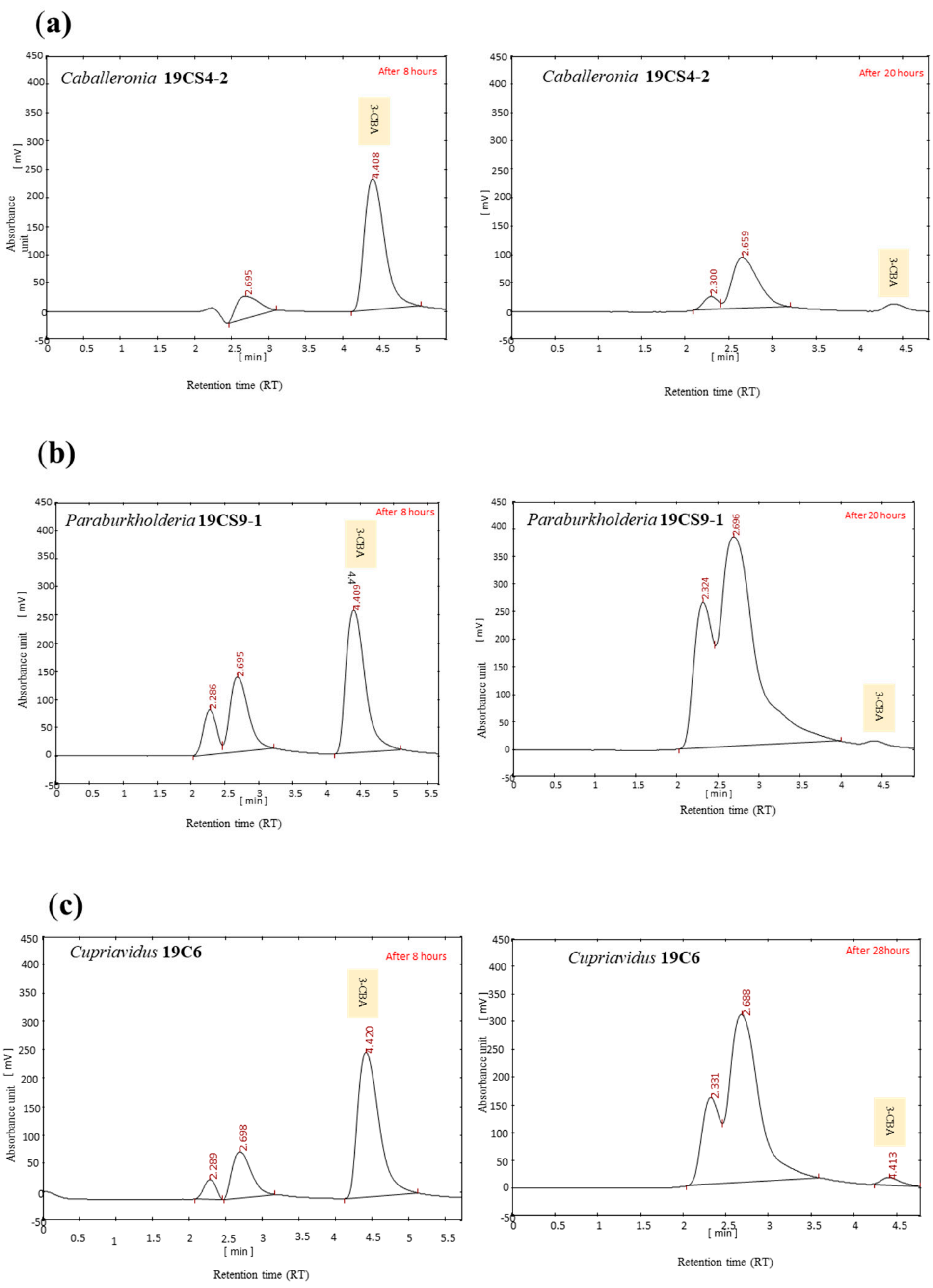

Figure S3: High performance liquid chromatography **(**HPLC) chromatogram result of the 3-chlorobenzoate (3-CBA) concentration reduction by the isolates 19CS4-2(a),19CS9-1(b), and 19C6 (c), respectively. After 8 hour incubation, the 3-CBA detected at 4.4 retention time (showed in left-image) and completely reduced the 3-CBA concentration (showed in right-image) within 20 hours by 19CS4-2 and 19CS9-1, whereas within 28 hours by 19C6.

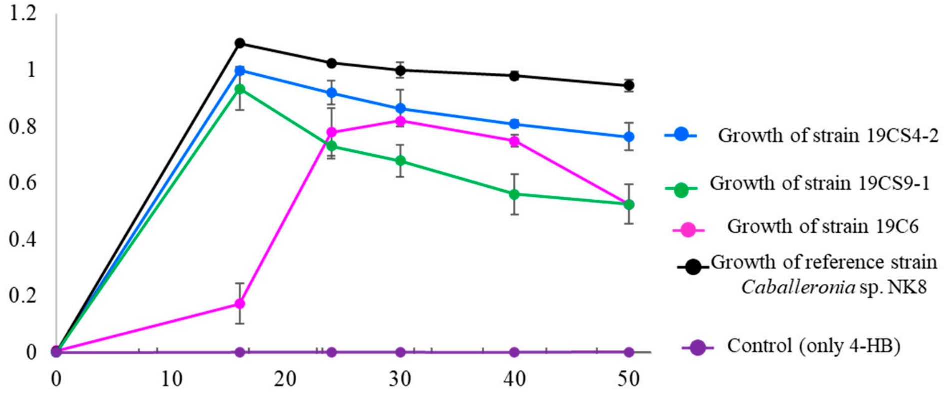

Figure S4: The cell growth of strains 19CS4-2,19CS9-1, and 19C6 in basal salt medium (BSM) supplemented with 5mM 4-hydrocybenzoate (4-HB). The data are represented as the means ± standard deviation for duplicate experiments. When the error bar is not visible, it lies within the data point.

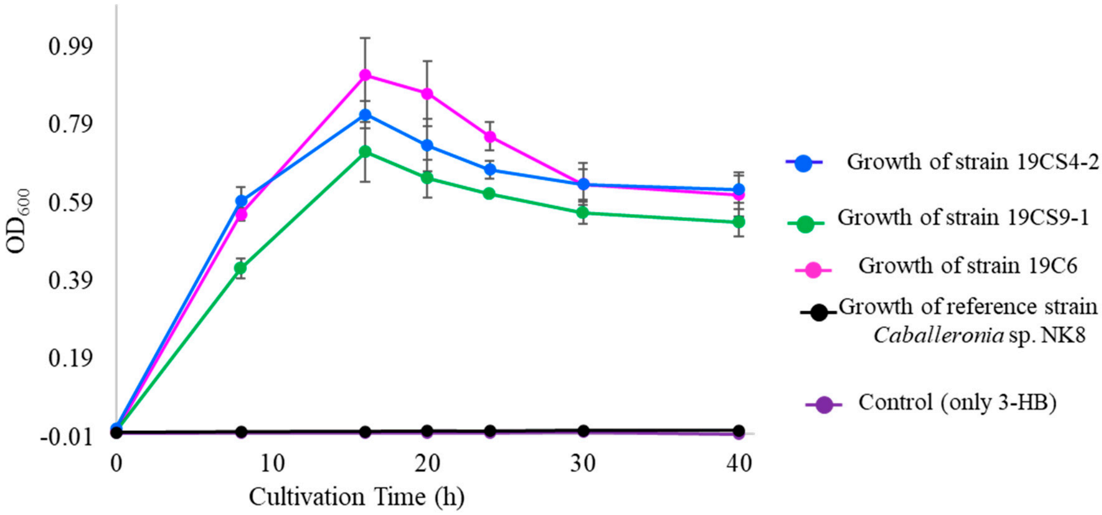

Figure S5: The cell growth of strains 19C6,19CS4-2 and 19CS9-1 in basal salt medium (BSM) supplemented with 5mM 3-hydrocybenzoate (3-HB). The data are represented as the means ± standard deviation for duplicate experiments. When the error bar is not visible, it lies within the data point.

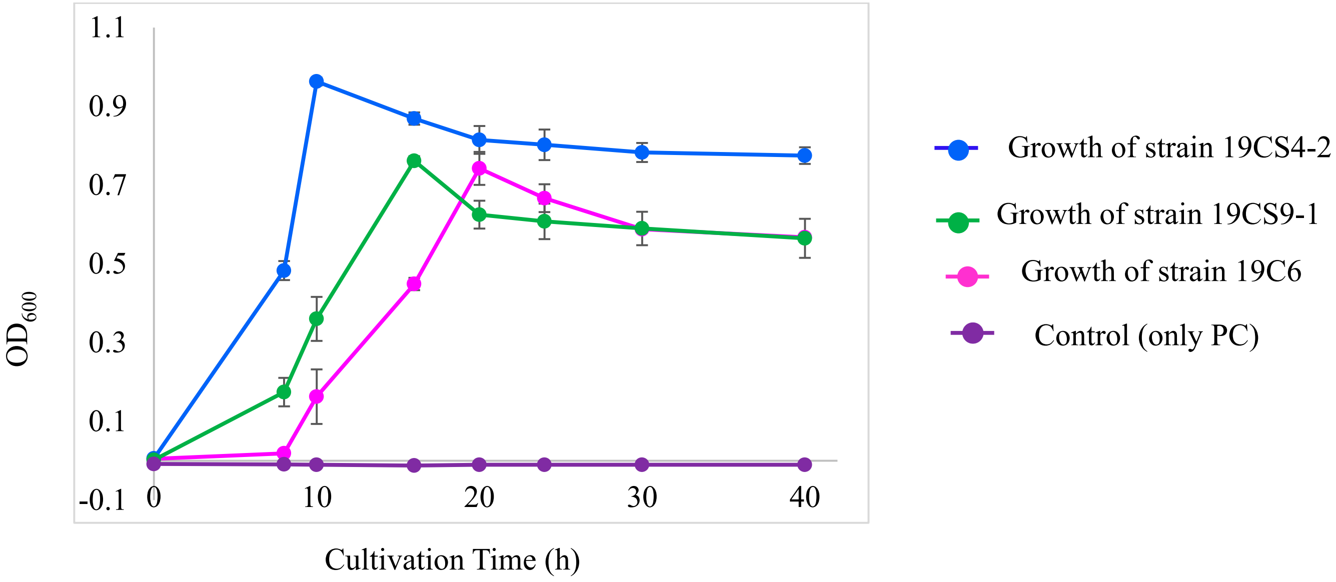

Figure S6: The cell growth of strains 19C6,19CS4-2 and 19CS9-1 basal salt medium (BSM) supplemented with 5mM protocatechuate (PC). The data are represented as the means ± standard deviation for duplicate experiments. When the error bar is not visible, it lies within the data point.

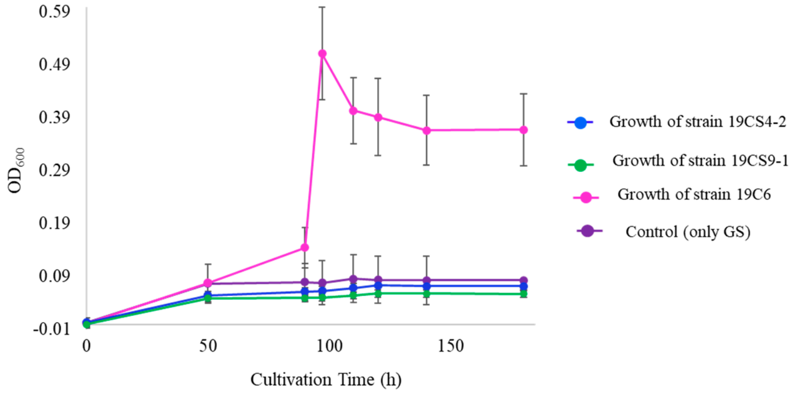

Figure S7: The cell growth of strains 19CS4-2,19CS9-1 and19C6, basal salt medium (BSM) supplemented with 5mM gentisate (GS). The data are represented as the means ± standard deviation for duplicate experiments. When the error bar is not visible, it lies within the data point.

Table S1: The values of Average amino acid identities (AAI) of the whole-genome DNA assembly sequences between the obtained isolates and closest strains in Genbank.

Isolates	Reference	AAI	#CDS (isolate)	#CDS (reference)	Matched count
**19CS4-2**	[a] LMG 28615(T)	93.3	8441	6263	5171
**19CS9-1**	[b] LMG 20598 (T)	95.0	8482	10409	6825
**19C6**	[c]CCUG 12507 (T)	86.9	6114	5952	4182

[a]*Caballeronia novacaledonica* LMG 28615 (T) (NZ_OGTP01000000)

[b] *Paraburkholderia hospita* LMG 20598 (T) (NZ_FNXA01000000)

[c]*Cupriavidus pauculus* CCUG 12507 (T) (NZ_VZOW01000000)

Table S2: The values of percentage of conserved protein (POCP) of the whole-genome DNA assembly sequences between the obtained isolates (19CS4-2,19CS9-1, and 19C6) and closest strains in Genbank (LMG 28615, LMG 20598*,* and CCUG 12507).

ID	19CS4-2	19CS9-1	19C6	LMG 28615(T)[a]	LMG 20598 (T)[b]	CCUG 12507 (T)[c]
**19CS4-2**	-	70.0	49.8	78.6	67.6	47.2
**19CS9-1**		-	52.2	68.8	82.0	49.6
**19C6**			-	52.0	49.3	75.5
**LMG 28615(T)[a]**				-	64.5	49.4
**LMG 20598 (T)[b]**					-	46.1

[a]*Caballeronia novacaledonica* LMG 28615 (T) (NZ_OGTP01000000)

[b] *Paraburkholderia hospita* LMG 20598 (T) (NZ_FNXA01000000)

[c]*Cupriavidus pauculus* CCUG 12507 (T) (NZ_VZOW01000000)

Table S3. The values of Average nucleotide identities between the obtained isolates (a) and reference strains (b).

Genome a	Genome b (Genbank Accession number)	Ortho ANIu value (%)	Genome A length (bp)	Genome B length (bp)	Average aligned length (bp)	Genome A coverage (%)	Genome B coverage (%)
**19CS4-2**	*Caballeronia novacaledonica* LMG 28615 (T) (NZ_OGTP01000000)	91.7	8,987,220	6,738,120	3,723,524	41.4	55.3
**19CS9-1**	*Paraburkholderia hospita* LMG 20598 (T) (NZ_FNXA01000000)	92.73	9,355,440	11,104,740	5,330,994	57.0	48.0
**19C6**	*Cupriavidus pauculus* CCUG 12507 (T) (NZ_VZOW01000000)	83.61	6,778,920	6,362,760	2,855,533	42.1	44.9

Table S4: Determination of the predicted genes involved in the strains 19CS4-2,19CS9-1 and 19C6 for 3-chlorobenzoate (3-CBA) degradation via chlorocatechol (CC) from the BlastKOALA results.

Gene[a]	K number[b]	Definition[a]	Locus tag	Contig No.	Reference strain (GenBank Accession)	Identity (%) nucleotide	Identity (%) amino-acid
Caballeronia sp. 19CS4-2 (3-CBA degradation)							
cbeE	K05782	(chloro)benzoate membrane transport protein	CBA19CS42_27660	15	*Caballeronia* sp. NK8 (AP024325)[d]	88	92
catC	K03464	muconolactone D-isomerase	CBA19CS42_27655	15	*Caballeronia* sp. NK8 (AP024325)[d]	89	95
catB	K01856	muconate cycloisomerase	CBA19CS42_27650	15	*Caballeronia* sp. NK8 (AP024325)[d]	90	94
cbeR	K21757	LysR family transcriptional regulator	CBA19CS42_27640	15	*Caballeronia* sp. NK8 (AP024325)[d]	89	94
catA	K03381	catechol 1,2-dioxygenase	CBA19CS42_27635	15	*Caballeronia* sp. NK8 (AP024325)[d]	90	93
cbeA	K05549	(chloro)benzoate 1,2-dioxygenase subunit alpha	CBA19CS42_27630	15	*Caballeronia* sp. NK8 (AP024325)[d]	94	96

cbeB	K05550	(chloro)benzoate 1,2-dioxygenase subunit beta	CBA19CS42_27625	15	*Caballeronia* sp. NK8 (AP024325)[d]	92	93
cbeC	K05784	(chloro)benzoate 1,2-dioxygenase reductase component	CBA19CS42_27620	15	*Caballeronia* sp. NK8 (AP024325)[d]	91	94
cbeD	K05783	dihydroxy-cyclohexadiene carboxylate dehydrogenase	CBA19CS42_27615	15	*Caballeronia* sp. NK8 (AP024325)[d]	89	91
tfdT	K05596	LysR family transcriptional regulator	CBA19CS42_31125	19	*Caballeronia* sp. NK8 (AP024328)[e]	100	100
tfdC	K15253	(chloro)catechol 1,2-dioxygenase	CBA19CS42_31120	19	*Caballeronia* sp. NK8 (AP024328)[e]	99	100
tfdD	K01860	(chloro)muconate cycloisomerase	CBA19CS42_3111 [c]	19	*Caballeronia* sp. NK8 (AP024328)[e]	100	100
tfdE	K01061	carboxymethylenebutenolidase	CBA19CS42_31110	19	*Caballeronia* sp. NK8 (AP024328)[e]	100	100
tfdF	K00217	maleylacetate reductase	CBA19CS42_31105	19	*Caballeronia* sp. NK8 (AP024328)[e]	100	100

Paraburkholderia sp. 19CS9-1 (3-CBA degradation)							
cbeR	K21757	LysR family transcriptional regulator	CBA19CS91_37010	42	*Caballeronia* sp. NK8 (AP024325)[d]	75	82
cbeA	K05549	(chloro)benzoate 1,2-dioxygenase subunit alpha	CBA19CS91_37005	42	*Caballeronia* sp. NK8 (AP024325)[d]	66	58
cbeB	K05550	(chloro)benzoate 1,2-dioxygenase subunit beta	CBA19CS91_37000	42	*Caballeronia* sp. NK8 (AP024325)[d]	64	52
cbeC	K05784	(chloro)benzoate 1,2-dioxygenase reductase component	CBA19CS91_36995	42	*Caballeronia* sp. NK8 (AP024325)[d]	62	53
cbeD	K05783	dihydroxy-cyclohexadiene carboxylate dehydrogenase	CBA19CS91_36990	42	*Caballeronia* sp. NK8 (AP024325)[d]	63	60
benM	K21757	LysR family transcriptional regulator	CBA19CS91_19860	11	*Caballeronia* sp. NK8 (AP024325)[d]	58	53
catB	K01856	muconate cycloisomerase	CBA19CS91_19865	11	*Caballeronia* sp. NK8 (AP024325)[d]	64	58

catA	K03381	catechol 1,2-dioxygenase	CBA19CS91_19870	11	*Caballeronia* sp. NK8 (AP024325)[d]	60	49
catC	K03464	muconolactone D-isomerase	CBA19CS91_19875	11	*Caballeronia* sp. NK8 (AP024325)[d]	65	54
tfdT	K05596	LysR family transcriptional regulator	CBA19CS91_38115	47	*Caballeronia* sp. NK8 (AP024328)[e]	83	86
tfdC	K15253	chlorocatechol 1,2-dioxygenase	CBA19CS91_38120	47	*Caballeronia* sp. NK8 (AP024328)[e]	86	90
tfdD	K01860	(chloro)muconate cycloisomerase	CBA19CS91_38125 [c]	47	*Caballeronia* sp. NK8 (AP024328)[e]	86	93
tfdE	K01061	carboxymethylenebutenolidase	CBA19CS91_38130	47	*Caballeronia* sp. NK8 (AP024328)[e]	85	93
tfdF	K00217	maleylacetate reductase	CBA19CS91_38135	47	*Caballeronia* sp. NK8 (AP024328)[e]	81	82
Cupriavidus sp. 19C6 (3-CBA degradation)							
cbeR	K21757	LysR family transcriptional regulator	CBA19C6_10295	4	*Caballeronia* sp. NK8 (AP024325)[d]	80	90

catA	K03381	catechol 1,2-dioxygenase	CBA19C6_10300	4	*Caballeronia* sp. NK8 (AP024325)[d]	86	89
cbeA	K05549	(chloro)benzoate 1,2-dioxygenase subunit alpha	CBA19C6_10305	4	*Caballeronia* sp. NK8 (AP024325)[d]	86	86
cbeB	K05550	(chloro)benzoate 1,2-dioxygenase subunit beta	CBA19C6_10310	4	*Caballeronia* sp. NK8 (AP024325)[d]	79	79
cbeC	K05784	(chloro)benzoate 1,2-dioxygenase reductase component	CBA19C6_10315	4	*Caballeronia* sp. NK8 (AP024325)[d]	85	90
cbeD	K05783	dihydroxy-cyclohexadiene carboxylate dehydrogenase	CBA19C6_10320	4	*Caballeronia* sp. NK8 (AP024325) [d]	80	87
benM	K21757	LysR family transcriptional regulator	CBA19C6_25785	17	*Caballeronia* sp. NK8 (AP024325)[d]	58	49
catB	K01856	muconate cycloisomerase	CBA19C6_25790	17	*Caballeronia* sp. NK8 (AP024325)[d]	90	81
catC	K03464	muconolactone D-isomerase	CBA19C6_25795	17	*Caballeronia* sp. NK8 (AP024325)[d]	89	79

tfdE	K01061	carboxymethylenebu tenolidase	CBA19C6_18665	8	*Caballeronia* sp. NK8 (AP024328)[e]	32	19
tfdF	K00217	maleylacetate reductase	CBA19C6_19205	9	*Caballeronia* sp. NK8 (AP024328)[e]	53	49

[a]Gene designation and definition from KEGG annotation were manually modified

[b]Blast-Koala result could assign K number for each CDSs, the number in the database of molecular functions represented in terms of functional orthologs

[c]2nd best hit from Blast-Koala result

[d]*Caballeronia* sp. NK8 plasmid pNK81 DNA, complete sequence

[e]*Caballeronia* sp. NK8 plasmid pNK84 DNA, complete sequence

Table S5: Determination of the predicted genes involved in the strains 19CS4-2,19CS9-1 and 19C6 for 4-HB degradation via protocatechuate (PC) and only in 19C6 for 3-HB degradation gentisate (GS), from the BlastKOALA results.

Gene[a]	K number[b]	Definition[a]	Locus tag	Contig No.	Reference strain (GenBank Accession)	identity (%) nucleotide	identity (%) aminoacid
		Caballeronia sp. 19CS4-2 (4-HB degradation)					
pobA	K00481	4-hydroxy-benzoate 3-monooxygenase	CBA19CS42_22240	9	*Caballeronia* sp. NK8 (AP024323)[c]	94	15
pcaG	K00448	protocatechuate 3,4-dioxygenase, alpha subunit	CBA19CS42_35245	28	*Caballeronia* sp. NK8 (AP024323)[c]	96	98
pcaH	K00449	protocatechuate 3,4-dioxygenase, beta subunit	CBA19CS42_35250	28	*Caballeronia* sp. NK8 (AP024323)[c]	95	98
pcaB	K01857	3-carboxy-*cis*, *cis*-muconate cycloisomerase	CBA19CS42_06185	1	*Caballeronia* sp. NK8 (AP024322)[d]	92	94
pcaC	K14727	4-carboxy-muconolactone decarboxylase	CBA19CS42_06195	1	*Caballeronia* sp. NK8 (AP024322)[d]	93	98
pcaD	K01055	3-oxoadipate enol-lactonase	CBA19CS42_06190	1	*Caballeronia* sp. NK8 (AP024322)[d]	92	95
pcaR	K02623	IclR family transcriptional regulator	CBA19CS42_06165	1	*Caballeronia* sp. NK8 (AP024322)[d]	90	97

pcaI	K01031	3-oxoadipate CoA-transferase, alpha subunit	CBA19CS42_06170	1	*Caballeronia* sp. NK8 (AP024322)[d]	95	97
pcaJ	K01032	3-oxoadipate CoA-transferase, beta subunit	CBA19CS42_06175	1	*Caballeronia* sp. NK8 (AP024322)[d]	93	95
pcaF	K00632	3-oxoadipyl-CoA thiolase	CBA19CS42_06180	1	*Caballeronia* sp. NK8 (AP024322)[d]	94	97
Paraburkholderia sp. 19CS9-1 (4-HB degradation)							
pobA	K00481	4-hydroxy-benzoate 3-monooxygenase	CBA19CS91_41520	70	*Caballeronia* sp. NK8 (AP024323)[c]	65	57
pcaG	K00448	protocatechuate 3,4-dioxygenase, alpha subunit	CBA19CS91_33170	31	*Caballeronia* sp. NK8 (AP024323)[c]	72	67
pcaH	K00449	protocatechuate 3,4-dioxygenase, beta subunit	CBA19CS91_33175	31	*Caballeronia* sp. NK8 (AP024323)[c]	83	82
pcaB	K01857	3-carboxy- *cis*, *cis*-muconate cycloisomerase	CBA19CS91_41535	70	*Caballeronia* sp. NK8 (AP024322)[d]	74	71
pcaC	K14727	4-carboxy	CBA19CS91_41545	70	*Caballeronia* sp. NK8 (AP024322)[d]	78	83

		muconolactone decarboxylase					
pcaD	K01055	3-oxoadipate enol-lactonase	CBA19CS91_41540	70	*Caballeronia* sp. NK8 (AP024322)[d]	70	64
pcaI	K01031	3-oxoadipate CoA-transferase, alpha subunit	CBA19CS91_41525	70	*Caballeronia* sp. NK8 (AP024322)[d]	88	91
pcaJ	K01032	3-oxoadipate CoA-transferase, beta subunit	CBA19CS91_41530	70	*Caballeronia* sp. NK8 (AP024322)[d]	79	80
pcaF	K00632	3-oxoadipyl-CoA thiolase	CBA19CS91_40720	61	*Caballeronia* sp. NK8 (AP024322)[d]	77	78
Cupriavidus sp. 19C6 (4-HB degradation)							
pobA	K00481	4-hydroxy-benzoate 3-monooxygenase	CBA19C6_22170	12	*Caballeronia* sp. NK8 (AP024323)[c]	60	50
pcaG	K00448	protocatechuate 3,4-dioxygenase, alpha subunit	CBA19C6_22185	12	*Caballeronia* sp. NK8 (AP024323)[c]	52	39
pcaH	K00449	protocatechuate 3,4-dioxygenase, beta subunit	CBA19C6_22190	12	*Caballeronia* sp. NK8 (AP024323)[c]	66	58
pcaB	K01857	3-carboxy- *cis*, *cis*-muconate cycloisomerase	CBA19C6_22180	12	*Caballeronia* sp. NK8 (AP024322)[d]	62	57

pcaL	K01055	4-carboxy-muconolactone decarboxylase	CBA19C6_22175	12	*Caballeronia* sp. NK8 (AP024322)[d]	66	46
pcaD	K14727	3-oxoadipate enol-lactonase	CBA19C6_25505	16	*Caballeronia* sp. NK8 (AP024322)[d]	66	46
pcaI	K01031	3-oxoadipate CoA-transferase, alpha subunit	CBA19C6_08335	3	*Caballeronia* sp. NK8 (AP024322)[d]	76	77
pcaJ	K01032	3-oxoadipate CoA-transferase, beta subunit	CBA19C6_08330	3	*Caballeronia* sp. NK8 (AP024322)[d]	75	77
pcaF	K00632	3-oxoadipyl-CoA thiolase	CBA19C6_08325	3	*Caballeronia* sp. NK8 (AP024322)[d]	76	12
Cupriavidus sp. 19C6 (3-HB degradation)							
mhbM	K22270	3-hydroxy-benzoate 6-monooxygenase	CBA19C6_06480	3	*Cupriavidus necator* NH9 (CP017758)[e]	85	87
mhbD	K00450	gentisate 1,2-dioxygenase	CBA19C6_06465	3	*Cupriavidus necator* NH9 (CP017758)[e]	86	91
mhbH	K01801	maleylpyruvate isomerase	CBA19C6_06475	3	*Cupriavidus necator* NH9 (CP017758)[e]	80	82
mhbI	K16165	fumarylpyruvate hydrolase	CBA19C6_06470	3	*Cupriavidus necator* NH9 (CP017758)[e]	85	84

lysR-type	K05596	LysR family transcriptional regulator	CBA19C6_06485	3	*Cupriavidus necator* NH9 (CP017758)[e]	76	82

[a]Gene designation and definition from KEGG annotation were manually modified

[b]Blast-Koala result could assign K number for each CDSs, the number in the database of molecular functions represented in terms of functional orthologs[d]*Caballeronia* sp. NK8 plasmid pNK81 DNA, complete sequence

[c]*Caballeronia* sp. NK8 DNA, chromosome 2, complete sequence

[d]*Caballeronia* sp. NK8 DNA, chromosome 1, complete sequence

[e]*Cupriavidus necator* NH9 chromosome 2, complete sequence
